# Telomeric repeats in the commercial SB-1 vaccine facilitate viral integration and contribute to vaccine efficacy

**DOI:** 10.1038/s41541-024-00945-6

**Published:** 2024-08-21

**Authors:** Yu You, Ahmed M. Kheimar, Tereza Vychodil, Lisa Kossak, Mohammad A. Sabsabi, Andelé M. Conradie, Sanjay M. Reddy, Luca D. Bertzbach, Benedikt B. Kaufer

**Affiliations:** 1 Freie Universität Berlin, Institute of Virology, 14163 Berlin, Germany; 2 Department of Poultry Diseases, Faculty of Veterinary Medicine, Sohag University, 82524 Sohag, Egypt; 3 Texas A&M University, School of Veterinary Medicine & Biomedical Sciences, Department of Veterinary Pathobiology, College Station, TX 77843 USA; 4 Leibniz Institute of Virology (LIV), Department of Viral Transformation, 20251 Hamburg, Germany; 5 Freie Universität Berlin, Veterinary Centre for Resistance Research (TZR), 14163 Berlin, Germany

**Keywords:** Herpes virus, Tumour virus infections

## Abstract

Marek’s disease virus (MDV) integrates its genome into the telomeres of host chromosomes and causes fatal lymphomas in chickens. This integration is facilitated by telomeric repeat sequences (TMRs) at the ends of the viral genome, and is crucial for MDV-induced lymphomagenesis. The SB-1 vaccine virus is commonly used in commercial bivalent vaccines against MDV and also contains TMRs at its ends. Here, we demonstrate that SB-1 efficiently integrates its genome into the chromosomes of latently infected T cells. Deletion of the TMRs from the SB-1 genome did not affect virus replication, but severely impaired virus integration and genome maintenance in latently infected T cells and in chickens. Strikingly, the reduced integration and maintenance of latent SB-1 significantly impaired vaccine protection. Taken together, our data revealed that the TMRs facilitate SB-1 integration and that integration and/or maintenance of the latent viral genome is critical for vaccine protection.

## Introduction

Marek’s disease virus (MDV; Mardivirus gallidalpha2; GaAHV2) is an oncogenic alpha herpesvirus that poses a significant threat to the global poultry industry, leading to substantial economic losses. MDV infects chickens and causes neurological disorders, immunosuppression, and deadly T-cell lymphomas, resulting in high mortality rates in unvaccinated chickens^1,2^. Despite the availability of vaccines, MDV remains a threat as the virus can evolve towards greater virulence and overcome the protection provided by existing vaccines^2–4^. Upon primary infection, MDV establishes latency primarily in CD4 + T cells and integrates into the telomeres of latently infected cells. Integration is crucial for MDV-induced transformation and is facilitated by telomeric repeat arrays (TMRs) consisting of hexanucleotide repeats (TTAGGG)_n_ present at both ends of the linear MDV genome^5–7^. Recent data support that MDV integrates into the host genome through homology-directed recombination and is excised via a t-loop-mediated process during reactivation^8^.

Live-attenuated vaccines have been developed to combat MDV. The three main MDV vaccines globally are: (i) the turkey herpesvirus (HVT; Mardivirus meleagridalpha1; MeAHV1), (ii) the attenuated GaAHV2 strain CVI988/Rispens, and (iii) the naturally apathogenic Mardivirus gallidalpha3 (GaAHV3) strain SB-1^2,9,10^. SB-1 was initially introduced in the mid-1980s and offers (in combination with the HVT vaccine) efficient protection against very virulent MDV strains. In various countries, including the USA, commercial SB-1/HVT bivalent vaccines, as well as CVI988/Rispens, are extensively used^11^ and facilitate the protection of billions of chickens. In addition, there are various groups developing SB-1-based vector vaccines^12^, which could complement the portfolio of commonly used HVT-based vector vaccines. These live-attenuated MDV vaccines possess large DNA genomes with a comparable genome structure and relatively high antigenic similarity to virulent MDV^13^. Intriguingly, they also harbor TMRs at the end of their genomes; however, their roles remain largely elusive^5,14,15^.

In this study, we investigated the integration of the SB-1 vaccine and the role of the TMRs in vaccine latency and protection. Our data revealed that SB-1 efficiently integrates into the ends of host chromosomes. Integration is facilitated by the TMRs and is crucial for SB-1 genome maintenance, latency, and efficient vaccine protection against MDV.

## Methods

### Ethics statement

All animal work was conducted according to relevant international and national guidelines for care and the humane use of animals and was approved by the LAGeSo (Landesamt für Gesundheit und Soziales) Berlin, Germany (approval number G0294-17). Animals were humanely euthanized as follows. Small chickens (up to 4 weeks of age) were stunned by a firm blow to the head and then euthanized by cervical dislocation. Larger chickens (including final necropsy) were anesthetized using a combination of ketamine (40 mg/kg) and xylazine (5 mg/kg) via intramuscular injection into the breast muscle and subsequently euthanized by cervical dislocation. All animal experiments were conducted in a blinded manner to eliminate subjectivity.

### Cells

Chicken embryo cells (CEC) were generated from fertilized specific pathogen-free (SPF) VALO eggs (VALO BioMedia GmbH; Osterholz-Scharmbeck, Germany), following previously published methods^16^. CEC were cultured in Eagle’s minimal essential medium (PAN Biotech; Aidenbach, Germany), complemented with 1 to 10% fetal bovine serum (PAN Biotech) and 1% penicillin [100 U/mL]/streptomycin [100 µg/mL] (AppliChem; Darmstadt, Germany) at 37 °C and 5% CO_2_. The reticuloendotheliosis virus (REV)-transformed chicken T cell line 855-19^17,18^, was cultured in RPMI 1640 (PAN Biotech; Aidenbach, Germany) supplemented with 1% sodium pyruvate (PAN Biotech), 1% non-essential amino acids (Biochrom; Berlin, Germany), 10% fetal bovine serum and antibiotics, and maintained at 41 °C in a 5% CO_2_ atmosphere.

### Viruses

**Fig. 1:**
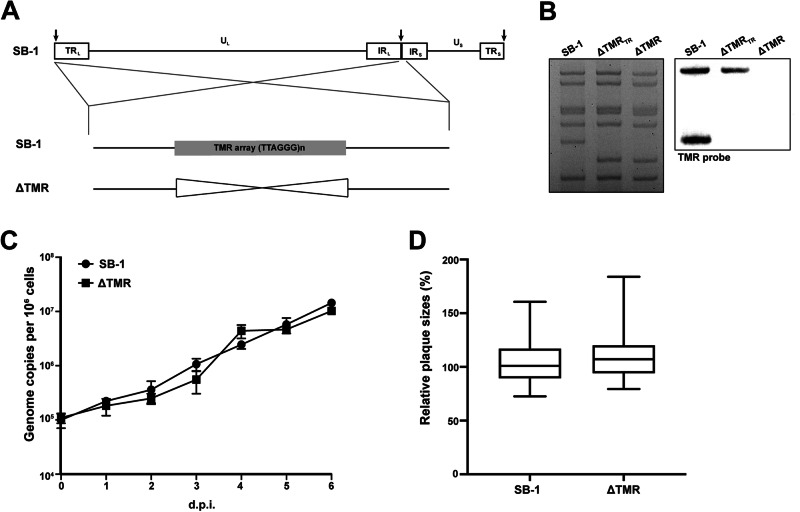
Generation and characterization of the ΔTMR mutant. **A** Schematic representation of the SB-1 genome with the TMR deletion in the terminal and internal repeat regions, resulting in the SB-1 ΔTMR mutant. **B** Restriction fragment length polymorphism patterns of the indicated viruses with the corresponding Southern blot analysis. The parental SB-1 BAC, the ΔTMR_TR_, and the double deletion mutant ΔTMR were digested with *HindIII*. The TMR sequences of the mutant viruses were detected using TMR-specific DIG-labeled probes. **C** Multi-step growth kinetics assays of indicated viruses. The viral genome copy numbers were quantified by qPCR. The mean copy numbers per 1 × 10^6^ cells are presented as the averages of three independent experiments (shown as means ± standard deviations; *p* > 0.05, Mann–Whitney *U*-test). **D** Plaque-size assays of indicated viruses. The box plots depict the mean plaque diameters from three independent experiments, with the minimum and maximum values. Statistical analysis (*p* > 0.05, Student’s *t*-test) was performed with a sample size greater than 50.

The GaHV-3 strain SB-1 lacking its TMRs was generated using the bacterial artificial chromosome (BAC) system of SB-1 published previously^19^. The mini-F cassette of the BAC contains an enhanced green fluorescent protein (eGFP) and was removed for the in vivo studies. The TMRs were deleted in the SB-1 genome using two-step Red-mediated recombination as described previously^20–22^. Briefly, the TMR copies in the SB-1 genome were sequentially deleted, resulting in a virus that lacked the TMRs in the terminal repeat region (∆TMR-TR) and one that lacked both copies (∆TMR; Fig. 1A). The resulting clones were confirmed by restriction fragment length polymorphism (RFLP), Southern blotting, and Sanger sequencing. In addition, the viruses used for in vivo experiments were verified by Illumina MiSeq sequencing with more than 1000-fold coverage. This deep coverage ensures that both the parental and mutant viruses are identical, except for the intended deletion of the TMRs. Recombinant viruses were reconstituted by transfection of CEC with purified BAC DNA using calcium phosphate transfection, as described previously^23^. All viruses were propagated in fresh CEC (max 3–5 passages). Virus stocks were frozen in liquid nitrogen and titrated prior to their use.

### Southern blotting

To verify the deletion of the TMRs, BAC DNA was digested with *HindIII* and then separated by agarose gel electrophoresis. The digested BAC DNA was transferred to a positively charged nylon membrane (Immobilon-NY+, Merck Millipore, Darmstadt, Germany) for Southern blot analysis. Fragments containing TMR arrays (Fig. 1B) were detected with a TMR-specific DIG-labeled probe (Table 1). The blots were subjected to immunological detection using an anti-DIG alkaline phosphatase-labeled antibody (Roche GmbH, Mannheim, Germany) followed by treatment with CDP-Star, ready-to-use (Roche GmbH), a chemiluminescent substrate for alkaline phosphatase.

**Table 1:** Primers and probes used in this study

Primer		Sequence (5′→3′)
ΔTMR (mutagenesis)	for	CCTTTTTTGGGGGGGGGGTGAAATGCAGGGGGGGATATTAAGTTGTGAATTTTTTTTATTCAGTTCTAGGGATAACAGGGTAATCGATTT
	rev	CCAAACGTCATACCAAAACTCTCGCGGCGGCGAACTGAATAAAAAAAATTCACAACTTAATATCCCCGCCAGTGTTACAACCAATTAACC
FISH PCR probe 1	for	GAGAAGAGCTCGAGTTGGTG
	rev	ACGAGCCGCTTGTAATTGAT
FISH PCR probe 2	for	TTACAACAGGAGGTTGGCAC
	rev	GATTTCGCTTCTTCATGGCG
FISH PCR probe 3	for	TCATACCAAAACTCTCGCGG
	rev	ATTAAGGGTAGCGGCTTTGG
FISH PCR probe 4	for	TCACGCCCACCACAAAAATA
	rev	CTGTACTCCGAACTGCTTCC
FISH PCR probe 5	for	GACCACGTATCCCTTATCGC
	rev	GCCTTTGGCGATTCTAGTCA
FISH PCR probe 6	for	CGACATCGCTCCAAAAGAGA
	rev	GTAGGGATCGGCTCAGTAGT
686_ICP4 (qPCR)	for	CGTGTTTTCCGGCATGTG
	rev	TCCCATACCAATCCTCATCCA
	probe	FAM-CCCCCACCAGGTGCAGGCA-TAM
SB-1_ICP4 (qPCR)	for	AATTTGCCACCACACCTCTTG
	rev	ATCACCGTCCTCGGAAACTG
	probe	FAM-GTCGAGGTCATCCGGCGGTGGCGGCGCAG-TAM
Chicken iNOS (qPCR)	for	GAGTGGTTTAAGGAGTTGGATCTGA
	rev	TTCCAGACCTCCCACCTCAA
	probe	FAM-CTCTGCCTGCTGTTGCCAACATGC-TAM

*for* forward primer, *rev* reverse primer, *FAM* 6-carboxyfluorescein, *TAM* TAMRA.

### Multi-step growth kinetics

The replication properties of the viruses were assessed by quantitative PCR (qPCR)-based multi-step growth kinetics as previously described^22^. Briefly, one million CEC were infected with 100 plaque-forming units (pfu) of the respective viruses. Cells were harvested at indicated time points over the course of 5 days, followed by DNA extraction using the RTP DNA/RNA Virus Mini kit (Stratec; Berlin, Germany). MDV genome copies of three independent experiments were evaluated by qPCR. Primers and probes specific to SB-1 infected cell protein 4 (ICP4) and chicken inducible nitric oxide synthase (iNOS) are listed in Table 1. Virus genome copies were normalized against the chicken iNOS gene.

### Plaque-size assays

To assess the cell-to-cell spread of the recombinant viruses, we performed plaque-size assays as described previously^22^. Briefly, one million CEC were infected with 100 pfu of each virus. Plaque areas were measured at 6 days post-infection (dpi) using the Bioreader (Bio-Sys; Karben, Germany), and plaque diameters were determined with the Bioreader software. Plaque-size assays were performed as three independent experiments.

### In vitro integration assays

To determine the integration efficiency of SB-1, we established an in vitro integration assay based on an assay developed for MDV^24^ using the chicken T cell line 855-19. One million cells were infected by co-cultivation with a highly infected CEC monolayer for 16 h as previously described^25^. T cells were then carefully removed, seeded into a new plate, and cultured for 14 days. The percentage of infected T cells was measured by flow cytometry using the CytoFlex S flow cytometer (Beckman Coulter, Brea, CA, USA). To assess the role of the TMRs in SB-1 genome maintenance, viral genome copies were quantified by qPCR at 1 and 14 dpi relative to cellular genome copies using specific primers and a probe for SB-1 ICP4 and the cellular iNOS gene (Table 1). Integration of SB-1 was visualized in metaphase chromosomes at 14 dpi with an SB-1-specific probe by fluorescent in situ hybridization (FISH) as described previously^24,26^.

### In vivo characterization of the ∆TMR virus

#### Animal experiment 1

To investigate the role of TMRs in SB-1 latency and reactivation, one-day-old SPF VALO chickens (VALO BioMedia)^27^ were randomly distributed into two groups and housed separately. The chickens of each group were infected subcutaneously with 2000 pfu of either the SB-1 (*n* = 6) or ΔTMR (*n* = 6). Three infected chickens per group were sacrificed at 14 and 28 dpi, respectively, and thymus, spleen, and blood samples were collected from each chicken. The lymphocytes from the spleen of each chicken were isolated using Ficoll density gradient centrifugation. Furthermore, feathers were collected from the chickens to assess the shedding of the virus. In addition, dust samples (three 1 mg aliquots) were collected from the air filters in each room at indicated time points.

#### Animal experiment 2

To determine the role of TMRs in integration in vaccine-induced protection, one-day-old SPF VALO chickens were vaccinated subcutaneously with 2,000 pfu of SB-1 (*n* = 25) or ΔTMR (*n* = 25). Vaccinated chickens were challenged at 7 days post vaccination (dpv) by intra-abdominal inoculation of 2000 pfu of the BAC-derived very virulent plus (vv+) GaAHV2 686 strain^28^. Non-vaccinated chickens (*n* = 10) infected with GaAHV2 686 at day 7 of age were included as a control group. Whole blood samples were collected at 4, 7, 10, 14, 21, 28, and 35 dpi. The experiment was performed in a blinded manner to eliminate any subjectivity. Chickens were monitored daily for the onset of clinical symptoms. Once clinical signs were detected or at the termination of the experiment (at 91 dpv), chickens were humanely euthanized and examined for gross tumor lesions.

### Virus quantification in blood samples, tissues, feather follicles, and dust samples

To assess virus replication in vivo, DNA from whole blood samples was isolated using the NucleoSpin 96 Blood Core Kit (Macherey-Nagel; Düren, Germany) according to the manufacturer’s instructions. To evaluate the efficiency of the virus shedding, DNA was extracted through treatment of the feather pulp and dust with proteinase K at 55 °C overnight, followed by phenol:chloroform:isoamyl alcohol extraction and ethanol precipitation as described previously^29,30^. MDV genome copies were measured by qPCR as described above, using primers and probe sets that can differentiate between the MDV challenge virus and the SB-1 vaccine (Table 1).

### Statistical analyses

Statistical analyses were performed using GraphPad Prism v9 (GraphPad Software, Inc.; San Diego, CA, USA). All statistical tests can be found in the respective figure legends. Data were considered significant if *p* ≤ 0.05 (*p < 0.05; **p < 0.01; ***p < 0.001; ****p < 0.0001).

## Results

### Generation and characterization of the SB-1 ∆TMR mutant

To elucidate the role of the TMRs in SB-1 replication, dissemination, latency, and reactivation, the TMRs in the internal and terminal repeat regions were sequentially deleted in the SB-1 BAC (SB-1 ΔTMR; Fig. 1A). The mutant virus genomes were confirmed by PCR, Sanger sequencing, RFLP, Southern blotting (Fig. 1B and Supplementary Fig. 1), and Illumina MiSeq sequencing. Upon reconstitution, we assessed whether the TMRs play a role in SB-1 replication using multi-step growth kinetics and plaque-size assays. Growth kinetics in cultured cells revealed that deletion of the TMR sequences did not alter virus replication compared to the parental SB-1 virus (Fig. 1C). Plaque-size assays confirmed this observation (Fig. 1D), highlighting that the TMR sequences are dispensable for SB-1 replication in vitro.

### Deletion of the TMRs severely impairs SB-1 integration in chicken T cells

**Fig. 2:**
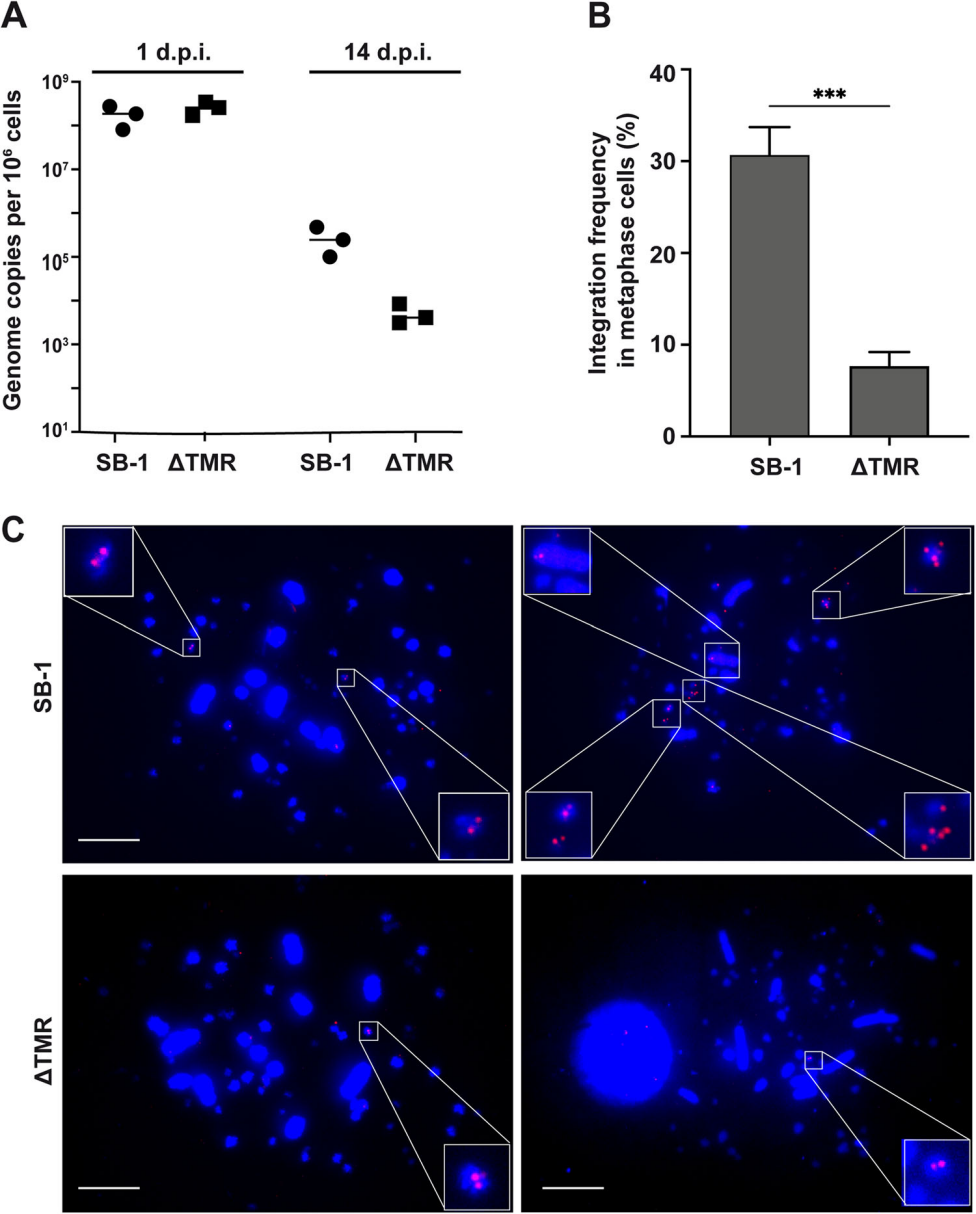
Genome maintenance and integration of SB-1 and the ΔTMR mutant in T cells. **A** Quantification of the maintenance SB-1 and ΔTMR in infected 855-19T cells. The persistence of the SB-1 genome in T cells was evaluated by qPCR at 1 and 14 dpi (*p* > 0.05, Mann–Whitney *U*-test, *n* = 3). **B** Integration frequency in metaphase cells was quantified by analyzing the integration status of 100 metaphases (***p < 0.001, Mann–Whitney *U*-test). Results are presented as means ± standard deviations, derived from three independent experiments. **C** Representative metaphase chromosomes (DAPI stain, blue) harboring the integrated virus (Cy3 streptavidin, red) in 855-19T cells infected with SB-1 (upper row) and SB-1 ΔTMR (lower row). Scale bars correspond to 10 μm.

Next, we established an in vitro integration assay to assess the integration efficiency of SB-1 and ΔTMR using 855-19T cells. Briefly, 855-19T cells were infected with the respective viruses. Integration and genome maintenance were monitored over time, as previously described for MDV^24^. While the initial infection levels were comparable between the viruses, genome maintenance was severely impaired (~55-fold) in the absence of the TMRs at 14 dpi (Fig. 2A, not significant (n.s.)). To determine if this is due to a defect in integration, we performed metaphase FISH analyses. Importantly, wild-type SB-1 efficiently integrated into the ends of one or multiple host chromosomes (Fig. 2B, C), indicating that this is the main mode of genome maintenance during latency. In contrast, the integration of ΔTMR was severely reduced (Fig. 2B, *p* < 0.001). Notably, the few integration events of the ΔTMR mutant virus were mostly not detected at the ends of the host chromosomes (Fig. 2C). Taken together, our data revealed that SB-1 efficiently integrates into the ends of host chromosomes and that the TMRs are important for integration and genome maintenance in latently infected cells.

### TMRs play a crucial role in SB-1 latency, transport to the skin, shedding, and reactivation in the host

**Fig. 3:**
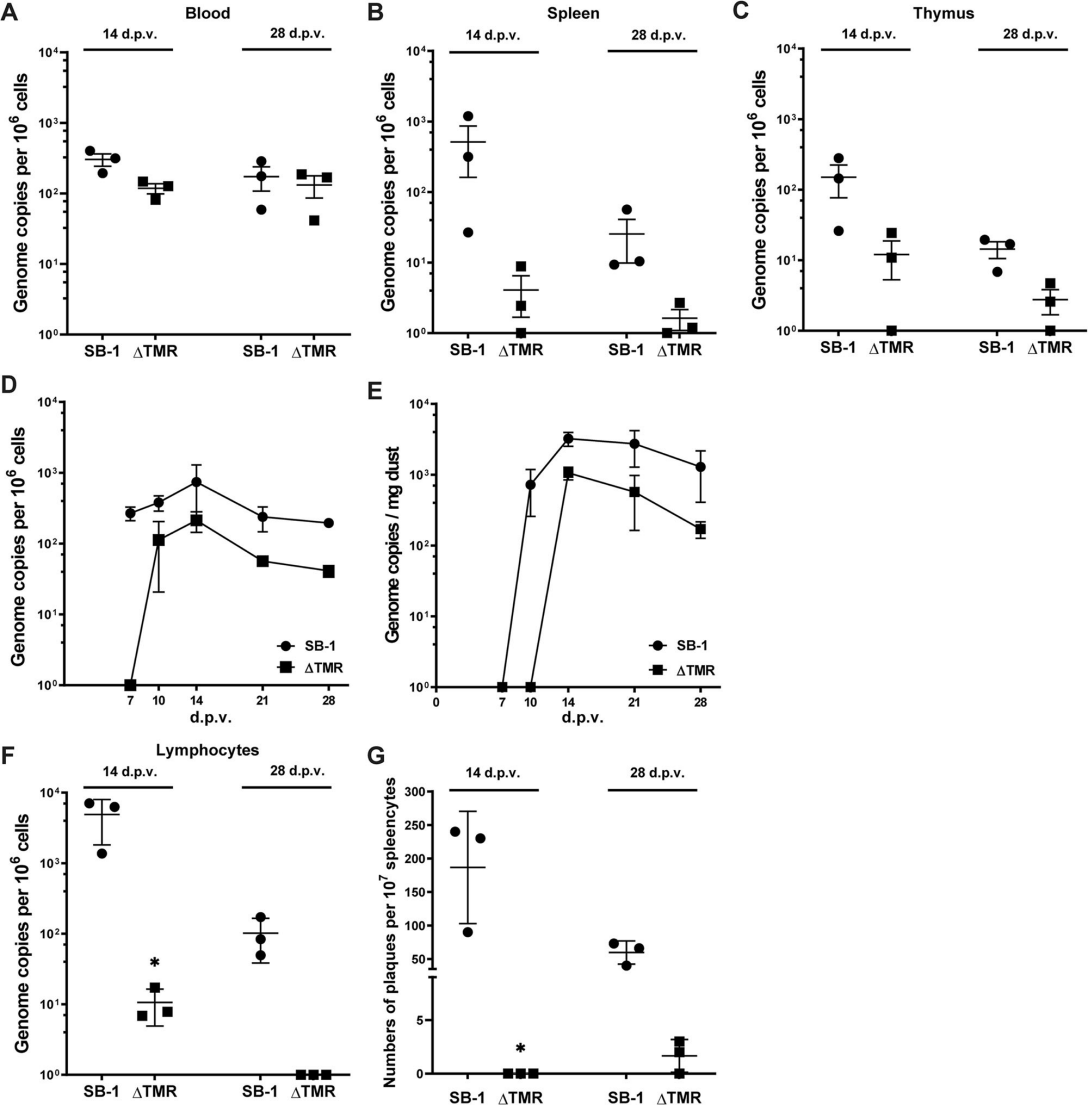
In vivo genome maintenance and reactivation of SB-1 and SB-1 ΔTMR. **A**–**C** Quantification of SB-1 genome copies in the blood (**A**), spleen (**B**), and thymus (**C**) by qPCR at 14 and 28 dpv. **D** Quantification of the genome copies of the indicated viruses in feather samples (pools of ten feathers from three chickens per group). **E** Viral copies per mg of dust (three 1 mg aliquots from the air filters in each room per time point) are shown for each group, as previously validated^35^. **F** Viral genome copies in purified lymphocytes from spleens of vaccinated chickens. **G** Reactivation of the indicated viruses, assessed by co-cultivation of 10^7^ purified lymphocytes from spleens of vaccinated chickens with fresh CECs, followed by plaque counting after 4 days of infection. **A**–**C**, **F**, **G**: Data points are displayed as dots, with the means represented by horizontal bars ± standard deviations (*p < 0.05, Mann–Whitney *U*-test, *n* = 3). **D**, **E** Data were shown as means ± standard deviations (*p* > 0.05, Mann–Whitney *U*-test).

To investigate if the observed integration defect affects SB-1 replication, genome maintenance, and latency in vivo, one-day-old chickens were infected with 2000 pfu of the ∆TMR or parental SB-1. First, we quantified viral genome copies in the blood of infected chickens at 14 and 28 dpv (Fig. 3A). qPCR revealed only a modest reduction in viral replication in the absence of the TMRs, indicating that the TMRs are dispensable for replication in vivo. To elucidate the role of the TMRs in viral genome maintenance during latency, we quantified viral genome copies in the spleen (Fig. 3B) and thymus (Fig. 3C) of the infected chickens. Our analyses revealed a severe decrease in SB-1 genome levels in these immune organs in the absence of TMRs, indicating that SB-1 latency is severely reduced (n.s.). Since latently infected cells are thought to transport the virus to the skin, we assessed the viral levels in the feather follicles. Transport to the skin was delayed in the absence of the TMRs when compared to wild-type SB-1, as no ∆TMR virus was detected at 7 dpi (Fig. 3D, n.s.). Virus levels were also reduced in the feather follicles, consistent with the reduced latent pool in the lymphoid organs. This was also reflected by reduced ∆TMR levels in the dust shed by the animals into the environment (Fig. 3E). To assess if viral reactivation is affected in the absence of TMRs, we isolated lymphocytes from the spleens containing the latently infected cells. Consistently, a severe reduction in the SB-1 genome copies was detected in the absence of the TMRs (Fig. 3F). Next, 10^7^ purified lymphocytes were co-cultivated with CEC to assess the reactivation frequency of SB-1 and ∆TMR. No, or hardly any ∆TMR reactivation was observed in cells harvested at 14 and 28 dpv, respectively (Fig. 3G). Taken together, these data suggest that SB-1 efficiently replicates in the absence of the TMRs, but that the levels of latency, transport to the skin, shedding, and reactivation are severely impaired.

### TMRs are crucial for vaccine protection

**Fig. 4:**
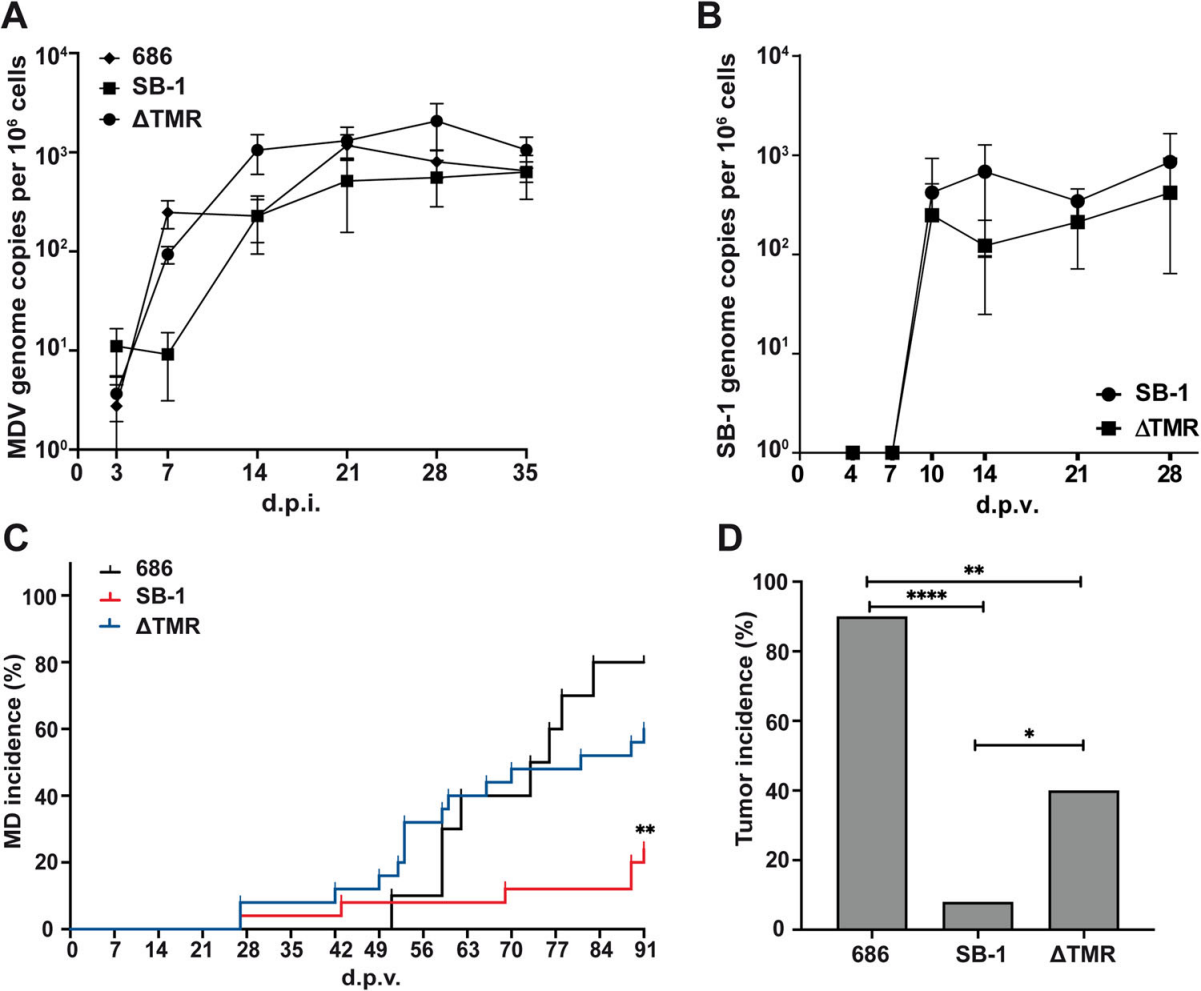
Replication and pathogenesis of the vv + GaAHV2 686 virus in chickens vaccinated with SB-1 or ΔTMR. **A** Quantification of MDV genome copies of 686 in the blood of mock (*n* = 10), SB-1 (*n* = 25), or ΔTMR vaccinated (*n* = 25) challenged with the 686 virus. Data were shown as means ± standard deviations. No statistically significant difference was observed (*p* > 0.05, Mann–Whitney *U*-test, *n* = 8 chickens were sampled per group and per time point). **B** Quantification of SB-1 and ΔTMR virus levels in the blood of these chickens (*n* = 8 animals per group and per time point). Data were shown as means ± standard deviations (*p* > 0.05, Mann–Whitney *U*-test). **C** Disease incidence in mock, SB-1, or ΔTMR vaccinated challenged with the 686 virus throughout the 91-day experiment (Mantel–Cox analysis; **p < 0.01). **D** Tumor incidence is shown as the percentage of tumor-containing chickens for the indicated groups. Statistical significance was determined using the Fisher’s exact test (*p < 0.05; **p < 0.01; ****p < 0.0001).

To investigate the role of the TMRs (and, in turn, integration) in vaccine protection, we vaccinated one-day-old chickens with SB-1 (*n* = 25), ∆TMR (*n* = 25), or mock (*n* = 10) and challenged them with the vv+ GaAHV2 strain 686 at 7 dpv. Animals were monitored for clinical symptoms for 91 dpv. First, we assessed the replication of the challenge virus and found that the vv+ strain efficiently replicated in all three groups (Fig. 4A), as published previously^28^. To ensure that the SB-1 vaccines were also efficiently replicated in these animals, we assessed the viral load in the blood by qPCR. Replication of the ∆TMR mutant was only slightly reduced compared to wild-type SB-1 (Fig. 4B), as observed in the first animal experiment (Fig. 3A). Disease incidence was efficiently reduced by the parental SB-1 vaccine (24%) compared to the mock-vaccinated chickens (80%). Strikingly, SB-1 vaccine protection was severely reduced in the absence of the TMRs (60% disease incidence; Fig. 4C, *p* < 0.01), indicating that integration, latency, and/or reactivation play important roles in vaccine protection. In addition, tumor incidence was very low in the SB-1-vaccinated group (8%), while 90% of the mock-vaccinated chickens developed tumors. In the absence of the TMRs, protection against tumors was severely impaired in the ∆TMR-vaccinated group (40%; Fig. 4D, *p* < 0.05). Taken together, our data revealed that vaccine protection is significantly reduced for ∆TMR, a virus that is severely impaired in integration, latency, and reactivation.

## Discussion

SB-1 is a component of bivalent MDV vaccines that are commonly used worldwide, and a significant number of chickens is vaccinated with it every year. To further understand the biology of this vaccine virus, we set out to investigate SB-1 integration and decipher the role of TMRs in infection as well as their influence on vaccine-induced protection. SB-1 can persist in the host for life, like all other herpesviruses. Most herpesviruses maintain their genome as a circular episome during latency. However, we and others previously demonstrated that the oncogenic MDV, HVT, as well as human herpesvirus 6 (HHV-6), can integrate their genomes into the telomeres of latently infected cells^7,14,15,31,32^. This ensures that the virus genome is efficiently maintained in latently infected cells, especially during cell proliferation. The integration process of MDV, HVT, and HHV-6 is facilitated by the TMRs present at the ends of the viral genomes^7,15,31^. These TMRs are identical to the telomere sequences (TTAGGG)_n_ found in all vertebrates, including humans and chickens^5,6^. Intriguingly, TMR sequences have been identified at the ends of 17 of the 83 full-length herpesvirus genomes, including SB-1^6^; however, their role remains elusive for almost all of them. Despite many decades of SB-1 being used as a vaccine, it remains unknown if the vaccine virus integrates into latently infected cells and if lifelong persistence plays an important role in vaccine protection. Since SB-1 also possesses TMRs at both ends of its genome, we set out to investigate SB-1 integration and the role of the TMRs in the life cycle of this important vaccine virus.

First, we generated SB-1 lacking its TMRs (∆TMR) and characterized the recombinant virus in vitro. Our data revealed that SB-1 efficiently replicates in the absence of its TMRs (Fig. 1C, D), as observed for HHV-6A, HVT, and MDV lacking their TMRs^7,15,31,32^. Next, we established an in vitro integration assay using chicken T-cells, based on the recently published system for MDV^24^. Importantly, SB-1 efficiently integrated into the ends of host chromosomes (Fig. 2A–C). A previous study tried to detect SB-1 integration in spleen cells of vaccinated animals ex vivo by FISH^14^. However, they did not detect latently infected cells harboring the integrated SB-1 genome, likely due to the very low number of latently infected cells in the spleen and other lymphoid organs. With our in vitro integration assay, we could dramatically increase the number of latently infected cells and show for the first time that SB-1 efficiently integrates into chicken T cells. In contrast to the wild-type virus, integration of SB-1 lacking the TMRs was not detected at the ends of the chromosomes in most cells. This is consistent with previous data that revealed that the related MDV can integrate randomly in the absence of its TMRs^7^. However, this integration is very inefficient compared to the TMR-mediated integration and occurs as genome concatemers. Random integration of DNA into host chromosomes has been observed in many studies, can be mediated by the DNA repair machinery, and occurs quite often in cell lines transfected with plasmids^33,34^. Overall, this data highlights that the TMRs are dispensable for SB-1 replication, but are crucial for efficient integration.

Next, we assessed the role of the TMRs in virus replication, latency, transport to the skin, shedding, and reactivation in the host. Our data revealed that SB-1 efficiently replicated in the blood of infected chickens and was only slightly reduced compared to wildtype (Figs. 3A, 4B), as previously observed for MDV and HVT lacking their TMRs^7,15^. To assess SB-1 latency, we investigated the viral levels in the spleen and thymus of infected animals. The viral loads detected in the spleen and thymus of ∆TMR-infected animals were severely reduced (Fig. 3B, C). This indicates that efficient integration of the viral genome chromosomes is required for efficient genome maintenance during latency in the host, as observed in T-cells in vitro. As latently infected cells are thought to transport the virus to the skin, where the virus is shed into the environment, we assessed SB-1 levels in feather samples. Strikingly, delivery to the skin was delayed in the absence of the TMRs (Fig. 3D). In addition, viral levels in the feathers at later time points were also reduced, suggesting that latently infected cells may contribute to dissemination to the skin. Consistently, virus shedding into the environment was also reduced (Fig. 3E). Denesvre and colleagues recently observed a similar phenotype for HVT lacking its TMRs^15^, highlighting that integration/latency is important for the shedding of these viruses. As MDV mutant viruses lacking the TMRs are severely impaired in their ability to reactivate^7^, we assessed the reactivation of SB-1 and ∆TMR. SB-1 reactivation was severely impaired in the absence of the TMRs (Fig. 3G). This SB-1 reactivation and re-exposure to the immune system could play an important role in vaccine efficacy, as a single SB-1 vaccine dose provides long-lasting protection^12^.

Finally, we assessed whether the TMRs (and, in turn, integration) contribute to SB-1 vaccine protection. Strikingly, vaccine protection was severely impaired in the absence of the TMRs (Fig. 4C). This could be due to the reduced latency and reactivation of the ∆TMR virus. Re-exposure to the immune system due to reactivation could be a contributing factor to the success of the SB-1 vaccine and will be assessed in future studies. Alternatively, differences in lytic replication of the SB-1 vaccine could also contribute to the reduced protection; however, virus replication was not (significantly) altered in vitro and in the blood of infected animals in the absence of the TMRs (Fig. 4B). Furthermore, protection against tumors was also impaired in the case of ∆TMR (Fig. 4D), as tumors are the main contributor to Marek’s disease.

In summary, our findings revealed that SB-1 efficiently integrates into the ends of host chromosomes, which ensures efficient maintenance in latently infected cells. The viral TMRs play a key role in SB-1 latency, transport to the skin, shedding, and reactivation of the virus. In the absence of the TMRs, SB-1 vaccine protection is severely impaired, highlighting the importance of virus integration, latency, and/or reactivation in its efficacy.

### Supplementary information

Supplementary Information

## Data Availability

The SB-1 and SB-1 ∆TMR sequences are available @GenBank with accession numbers PP982911 and PP982912, respectively.
