# Supplementary material for: Telomeric repeats in the commercial SB-1 vaccine facilitate viral integration and contribute to vaccine efficacy
Source: NPJ Vaccines. 2024 Aug 21;9:154. doi: 10.1038/s41541-024-00945-6 (PMC11339279; doi:10.1038/s41541-024-00945-6)
Supplement: Supplementary file 1 — Supplementary Information [file 41541_2024_945_MOESM1_ESM.pdf]

Supplementary Information

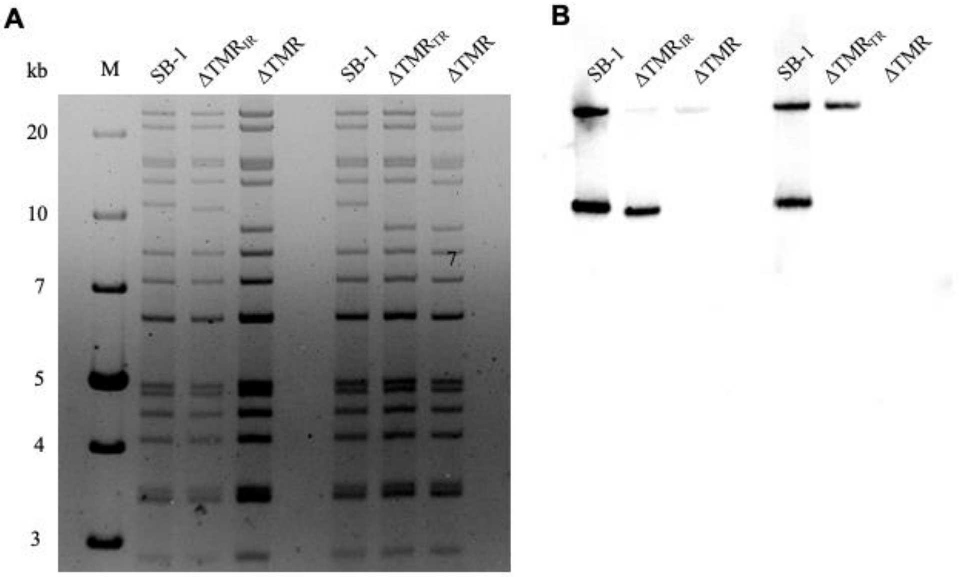

Supplementary Figure 1. Original images of restriction fragment length polymorphism patterns (A) and the corresponding Southern blot (B). The parental SB-1 BAC, the ΔTMR_{IR}, the ΔTMR_{TR}, and the double deletion mutant ΔTMR were digested with *HindIII*. The TMR sequences of the mutant viruses were detected using TMR-specific DIG-labeled probes.
